# Sputum immunoglobulin E levels correlate with eosinophilic airway regardless of atopy

**DOI:** 10.1186/s13223-025-00976-1

**Published:** 2025-06-30

**Authors:** Hyo-In Rhyou, Thi Bich Tra Cao, Quang Luu Quoc, Young-Hee Nam, Hae-Sim Park

**Affiliations:** 1 Department of Internal Medicine, Haeundae Paik Hospital, Inje University School of Medicine, Busan, Korea; 2 Department of Allergy and Clinical Immunology, Ajou University School of Medicine, 206 Worldcup-Ro, Yeongtong-Gu, Suwon, 16499 Korea; 3 Department of Biomedical Sciences, Ajou University School of Medicine, Suwon, Korea; 4 Department of Internal Medicine, College of Medicine, Dong-A University, Dong-A University Hospital, 26 Daesingongwon-Ro, Seo-Gu, Busan, 49201 Korea

**Keywords:** Asthma, Atopy, Immunoglobulin E, Eosinophil, Sputum, Serum, Eosinophil-derived neurotoxin, Anti-IgE antibody

## Abstract

Immunoglobulin E (IgE) is a key molecule that induces mast cell activation in allergic inflammation and contributes to type 2/eosinophilic inflammation in asthmatic airways. This cross-sectional study investigated the role of local IgE in asthmatic airways according to atopy, asthma control, and eosinophilic inflammation. A total of 31 adult patients with moderate-to-severe asthma were enrolled. The study subjects were classified into (1) atopic/non-atopic, (2) controlled/partly controlled/uncontrolled asthma and (3) eosinophilic/non-eosinophilic asthma. Serum/sputum IgE and serum/urine eosinophil-derived neurotoxin (EDN) were measured. Serum IgE levels were higher in atopic asthmatics than in non-atopic asthmatics, whereas no differences were noted in sputum IgE levels. Sputum IgE levels were significantly higher in uncontrolled asthmatics than in partly controlled or controlled asthmatics, and in eosinophilic asthmatics than in non-eosinophilic asthmatics, whereas no differences were noted in serum IgE levels. Significant correlations were observed between serum EDN and serum/sputum IgE levels. The production of local IgE in asthmatic airways could contribute to type 2/eosinophilic inflammation, irrespective of atopy, resulting in poor asthma control. Strategies targeting IgE may be effective in the management of non-atopic and atopic asthma.

Immunoglobulin E (IgE)-mediated processes have traditionally been associated with atopic asthma, and recent evidence suggests that IgE also plays a significant role in airway inflammation in non-atopic asthma [1]. Survival and cytokine release of mast cells may be affected by binding of IgE itself on mast cell surface irrespective of atopic status [2]. In addition, local IgE levels have been reported to be related to T2/eosinophilic inflammation independent of serum IgE levels and atopic status [3].

We conducted this study to explore the local and systemic production of IgE and its clinical relevance in asthma. A total of 31 adults with moderate to severe asthma who had been taking anti-asthmatic medications, including inhaled corticosteroids and long-acting beta2 agonists, at Ajou University Hospital (Suwon, Korea) participated in this study. Atopy was defined as a positive skin prick test reaction (ratio of the mean diameter of the wheal of the allergen to histamine ≥ 1) to 55 common aeroallergens (Bencard Co., Brentford, UK). Asthma and its control status were evaluated according to the Global Initiative for Asthma guidelines [4]. Patients with blood total eosinophil count ≥ 300 cells/µL were classified as eosinophilic asthma. Serum/sputum/urine samples were collected from each patient following established procedures [5]. Total IgE levels were measured using ImmunoCAP (ThermoFisher, Waltham, MA, USA) and eosinophil-derived neurotoxin (EDN) levels were measured using enzyme-linked immunosorbent assay (SKIMS-BIO Co., Seoul, Korea).

This study compared sputum and serum IgE levels between patients with atopic asthma (n = 15, 48.4%) and those with non-atopic asthma (n = 16, 51.6%). No significant differences were noted in the sputum IgE level between the two groups (*P* = 0.169; Fig. 1a), whereas significantly higher levels of serum IgE were observed in patients with atopic asthma than in those with non-atopic asthma (*P* = 0.004; Fig. 1b). No significant correlation was noted between sputum and serum IgE levels (*r* = 0.202, *P* = 0.284). Sputum IgE levels were significantly higher in the uncontrolled asthma group than in the controlled and partly controlled asthma groups (*P* = 0.021 and *P* = 0.019, respectively; Fig. 1c), whereas serum IgE levels were not significantly different among the three groups (*P* > 0.050; Fig. 1d). There were significant correlations between sputum IgE levels, asthma control test scores, asthma control questionnaire scores, and asthma quality of life questionnaire scores (*r* = −0.57, *P* < 0.001; *r* = 0.49, *P* = 0.006; and *r* = −0.48, *P* = 0.007, respectively). However, no significant association was found between sputum IgE levels and lung function parameters. Higher levels of sputum IgE, but not serum IgE, were noted in patients with eosinophilic asthma compared to those with non-eosinophilic asthma (*P* = 0.036 for sputum IgE; *P* = 0.074 for serum IgE; Fig. 1e and f). Moreover, significant positive correlations were noted between sputum IgE levels and urine/serum EDN levels (*r* = 0.400, *P* < 0.001 for urine and *r* = 0.500, *P* < 0.001 for serum).

**Fig. 1:**
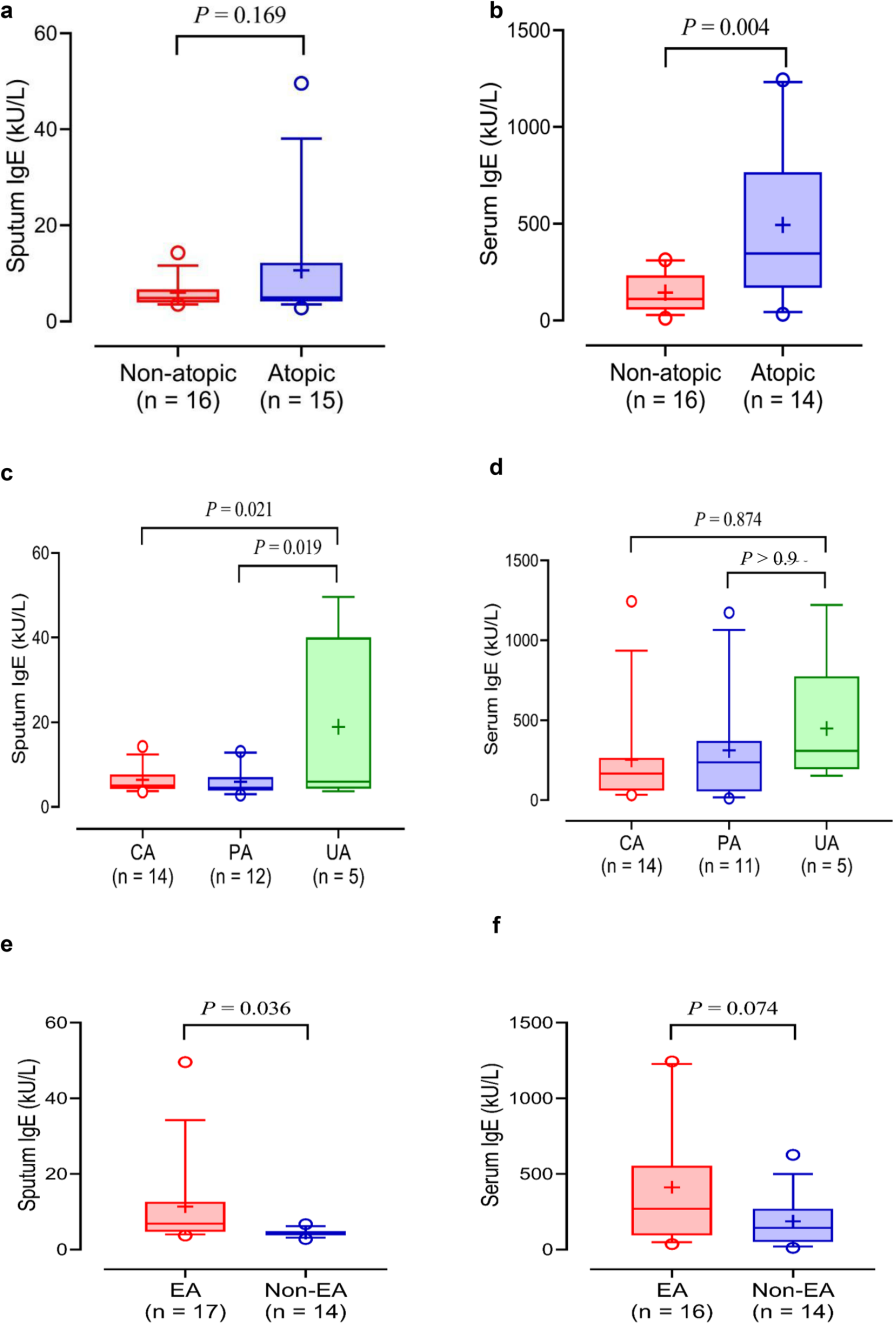
Comparison of sputum and serum total IgE levels according to atopic status, asthma control status, and phenotype of asthma. Comparisons of **a** sputum and **b** serum total IgE levels between patients with atopic asthma and those with non-atopic asthma. Comparison of **c** sputum and **d** serum IgE levels according to asthma control status in asthmatic subjects. Comparison of **e** sputum and **f** serum IgE levels between patients with eosinophilic asthma and those with non-eosinophilic asthma. Data are median (horizontal line), box edges are Q1-Q3, and whiskers are 10-90th percentile. *P* values were obtained by Student's t-test or one-way ANOVA with Bonferroni’s post hoc test. *CA* controlled asthma, *EA* eosinophilic asthma, *IgE* immunoglobulin E, *UA* uncontrolled asthma, *PA* partly controlled asthma

The local production of IgE in asthmatics was not associated with atopic status and did not correlate with serum IgE levels in the present study. Local IgE can be produced by stimulation of allergens, but increased local IgE levels are also present in non-atopic patients [3]. IL-4, IL-5, and IL-13 from innate lymphoid cells can induce IgE production of B cells without action of antigen-presenting cells and T cells in the airway [6]. A close association between sputum IgE levels, asthma control status, eosinophilic asthma phenotype, and EDN levels in the urine and serum (a marker for eosinophil degranulation) are confirmed in the current study, indicating that local IgE in airway secretions may contribute to T2/eosinophilic inflammation in asthmatics regardless of atopic status, and be related to asthma control status. Additionally, local IgE in asthmatic airways may contribute to eosinophilic airway inflammation. These findings suggest that the current anti-inflammatory medications, including inhaled corticosteroids, have limitations in modulating sputum IgE levels. Anti-IgE therapy is recommended for patients with severe allergic asthma but not for those with non-atopic asthma [4]. However, anti-IgE biologics also yield positive outcomes in patients with severe non-atopic asthma, although the mechanism is yet to be clarified [7]. Taken together, local IgE present in asthmatic airways could contribute to eosinophilic airway inflammation, and may be a therapeutic target in asthma regardless of atopic status.

This study has a few limitations. First, this is a single-center study with a relatively small sample size. Secondly, this is a cross-sectional study. A longitudinal study design would be beneficial to provide more comprehensive insights into the role of sputum IgE as a therapeutic target for T2 biologics in the management of severe asthma. Thirdly, the relationship between sputum IgE and additional biomarkers, such as sputum eosinophils, needs to be further explored.

In conclusion, local IgE present in asthmatic airways could contribute to eosinophilic airway inflammation, supporting the rationale that anti-IgE therapy could be effective in patients with non-atopic and atopic asthma.

## Data Availability

No datasets were generated or analysed during the current study.

## Abbreviations

IgE: Immunoglobulin E
EDN: Eosinophil-derived neurotoxin
